# Miniature Autonomy as Means to Find New Approaches in Reliable Autonomous Driving AI Method Design

**DOI:** 10.3389/fnbot.2022.846355

**Published:** 2022-07-01

**Authors:** Tim Tiedemann, Luk Schwalb, Markus Kasten, Robin Grotkasten, Stephan Pareigis

**Affiliations:** AutoSys Group, Department of Computer Science, Faculty TI, University of Applied Sciences (HAW), Hamburg, Germany

**Keywords:** autonomy, miniature autonomy, machine learning, evaluation, simulation, physical tests

## Abstract

Artificial Intelligence (AI) methods need to be evaluated thoroughly to ensure reliable behavior. In applications like autonomous driving, a complex environment with an uncountable number of different situations and conditions needs to be handled by a method whose behavior needs to be predictable. To accomplish this, simulations can be used as a first step. However, the physical world behaves differently, as the example of autonomous driving shows. There, erroneous behavior has been found in test drives that was not noticed in simulations. Errors were caused by conditions or situations that were not covered by the simulations (e.g., specific lighting conditions or other vehicle's behavior). However, the problem with real world testing of autonomous driving features is that critical conditions or situations occur very rarely—while the test effort is high. A solution can be the combination of physical world tests and simulations—and miniature vehicles as an intermediate step between both. With model cars (in a sufficiently complex model environment) advantages of both can be combined: (1) low test effort and a repeatable variation of conditions/situations as an advantage like in simulations and (2) (limited) physical world testing with unspecified and potentially unknown properties as an advantage like in real-world tests. Additionally, such physical tests can be carried out in less stable cases like already in the early stages of AI method testing and/or in approaches using online learning. Now, we propose to use a) miniature vehicles at a small scale of 1:87 and b) use sensors and computational power only on the vehicle itself. By this limitation, a further consequence is expected: Here, autonomy methods need to be optimized drastically or even redesigned from scratch. The resulting methods are supposed to be less complex—and, thus, again less error-prone. We call this approach “Miniature Autonomy” and apply it to the road, water, and aerial vehicles. In this article, we briefly describe a small test area we built (3 sqm.), a large test area used alternatively (1,545 sqm.), two last generation autonomous miniature vehicles (one road, one aerial vehicle), and an autonomous driving demo case demonstrating the application.

## 1. Introduction

One application field with a very high demand for trust and reliability on the one hand and a frequently chosen application of AI methods, on the other hand, is the field of advanced driver assistance systems (ADAS) and autonomous driving (AD). Usually, complex AI-based solutions are expected to be used (and proposed) for tasks in this area.

Machine learning (ML) based ADAS/AD solutions need to be evaluated thoroughly and usually with more effort than other methods since their correctness is more difficult to show (at least for approaches that do not follow the paradigm of “explainable AI (XAI)”). What needs to be verified is, first, the fulfillment of the specification and, second, the correctness of the specification. Mistakes can be made in the selection and collection of training and test data and the ML design (e.g., net architecture or training method).

Incorporating simulations can be a first step in evaluating ML-based ADAS/AD solutions. However, the physical world behaves differently, as the example of autonomous driving shows, where there are often unrecognized properties, conditions, circumstances, or situations that can lead to an erroneous behavior. Accidents of autonomous vehicles (AVs) have been analyzed in many studies, including comparisons between incidents caused by AV and such caused by humans (Wang et al., 2020; Ren et al., 2021). When taking a closer look at the accidents caused by AV, cases can be found where the actual error can be easily identified and fixed (e.g., misperception of a truck in camera data and/or a false action consequence when contradicting radar and camera perceptions are sensed). However, as long as the developers are not aware of the causing situation and conditions it might not be part of the simulation and, thus, it can not be identified in simulations. One main problem is the intersection of errors in the simulation test setup and the unknown unknowns (Hejase et al., 2020). In real-world tests, the possibility to introduce such errors in the test design is much more limited.

Now, evaluations in the real world usually require a lot of effort in setup and execution and, therefore, can only be carried out in a much more limited way than simulations. This contradicts the point that specific conditions, circumstances, and situations do usually occur only rarely (Tiedemann et al., 2019).

As a solution, we propose a method that we call “Miniature Autonomy” which is, first, a reduction of the physical model tests to a 1:87 scale and, second, a different design approach. We use a scale of 1:87 to be able to use (a) existing model train areas (refer to Section 2.1.2) and (b) off-the-shelf components for test areas and vehicles. The use of downscaled model vehicles is a common approach (Paull et al., 2017; Gerstmair et al., 2021), including competitions (Zug et al., 2014; Kuhnt et al., 2016; Carolo-Cup, 2021).

It reduces the effort required to set up and operate test areas, enables the variation of lighting conditions, environments, and traffic situations, allows testing around the clock, and reduces the risks of damage. The latter enables the application of early-stage designs and, in particular, unusual (e.g., complex biologically or cognitively inspired), online learning, end-to-end learning approaches, and dangerous, i.e., near-crash edge cases that are too risky to test in real traffic. Therefore, it is not important to mimic all real-world scenarios and driving conditions in the miniature model world but to be sufficiently complex to force the development of methods that can later be applied to real-world setups as well. Compared to simulation-only tests a reduction of needed real-world test miles is one goal. Another goal is to have a qualitative difference in mixed tests i.e., simulation and physical (model) tests (compared to simulation-only tests).

A further field of applications is autonomous aerial vehicles (e.g., drones) that are dangerous to test in real world environments. As a disadvantage, one might argue now that the model behaves differently than real 1:1 vehicles. Therefore, model car tests do not replace real traffic tests but could possibly reduce the number of test miles with real vehicles.

Furthermore, by the term “Miniature Autonomy” we propose a different approach to ADAS/AD solution design. For our 1:87 scale vehicles, autonomy covers energy consumption, actuation, sensors, and data processing including the whole autonomy under test. That is, the vehicles perform the sensor measurements, environment modeling, decision making, and the execution itself. To accomplish this, as expected, new solutions need to be found. In order to find a solution for the miniature vehicle, a new, much simpler solution must be developed which in turn should be less error-prone. It is not just scaling down existing approaches. The new solution is supposed to be transferable to a real 1:1 vehicle (Pareigis et al., 2019, 2021; Tiedemann et al., 2019).

Presented and briefly described are a small test area, the properties of a large test area, two vehicles (one road vehicle, one aerial vehicle), and a demo case for the application of the proposed AD design approach of “miniature autonomy.”

## 2. Materials and Equipment

The description of the proposed “miniature autonomy” design method is divided into two parts. First, the general model test approach is described, which consists of the model test areas used in our projects and two of the vehicles developed so far. Other vehicles that we have designed and implemented were a compact sedan car, other trucks, and a ship (Burau et al., 2019; Tiedemann et al., 2019). The second part describes the “miniature autonomy” design approach itself in Section 3 followed by a first demo case in Sections 4.2 and following. This first demo case is used as an “algorithm under test (AUT)” to evaluate the “miniature autonomy” approach.

### 2.1. Miniature Model World

Two different model environments are used for the miniature autonomy projects. The Miniatur Wunderland in Hamburg is a large model railroad for commercial purposes. It serves as a role model for our smaller *Micro-Wunderland*. The two environments are described in the following sections.

#### 2.1.1. Small Test Area

**Figure 1:**
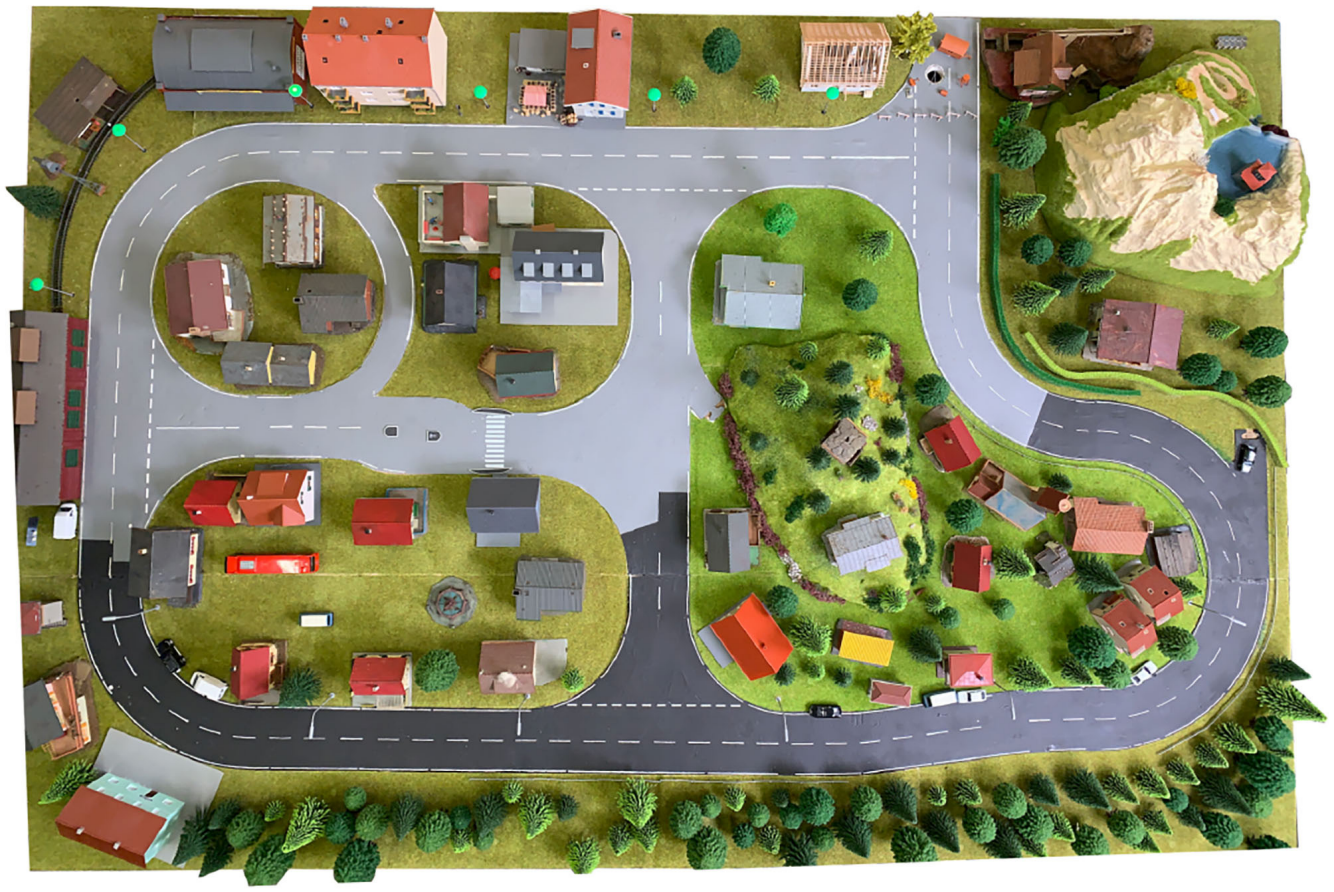
Bird's view of the small test area. Houses, trees, etc. are held with magnets to be easily movable. The image shows a typical placement of these objects as it was used for the first tests. Roadway surfaces and road markings have intentionally varying colors and quality.

The small test area, or *Micro-Wunderland*, is a 1:87 sized model city with roads for model cars. The area of the model city is approximately 2 × 1.5 m^2^. The *Micro-Wunderland* was built in our autosys research lab to create a realistic model environment for miniature autonomous vehicles. The roads have an integrated magnetic wire, which allows (automated but non-autonomous) model cars to drive along the wire, e.g., to generate environmental traffic. Switches under the roadway allow automated traffic to be rerouted. The model city has traffic lights, street lamps, and traffic signs that can be used by miniature autonomous vehicles in various ways. The houses and other objects are attached with magnets to allow a quick reconstruction of the environment. The road surface has different colors and varying road markings to better match the complexity of a real environment. Lighting conditions can be changed. The total cost of the Micro-Wunderland is in the order of 3,000 Euro. A top view is shown in Figure 1.

Two different localization methods were set up on the small test area (to serve as ground truth and to mimic GNSS-based localization): 1. a fixed camera in top-view position, combined e.g., with markers at the vehicles, and 2. ultra wide band (UWB) based radio localization, e.g., with trilateration. The small test area *Micro-Wunderland* is located in our research lab and is, therefore, easily accessible to allow easy generation of different kinds of data, especially training data for machine learning algorithms.

#### 2.1.2. Large Test Area

**Figure 2:**
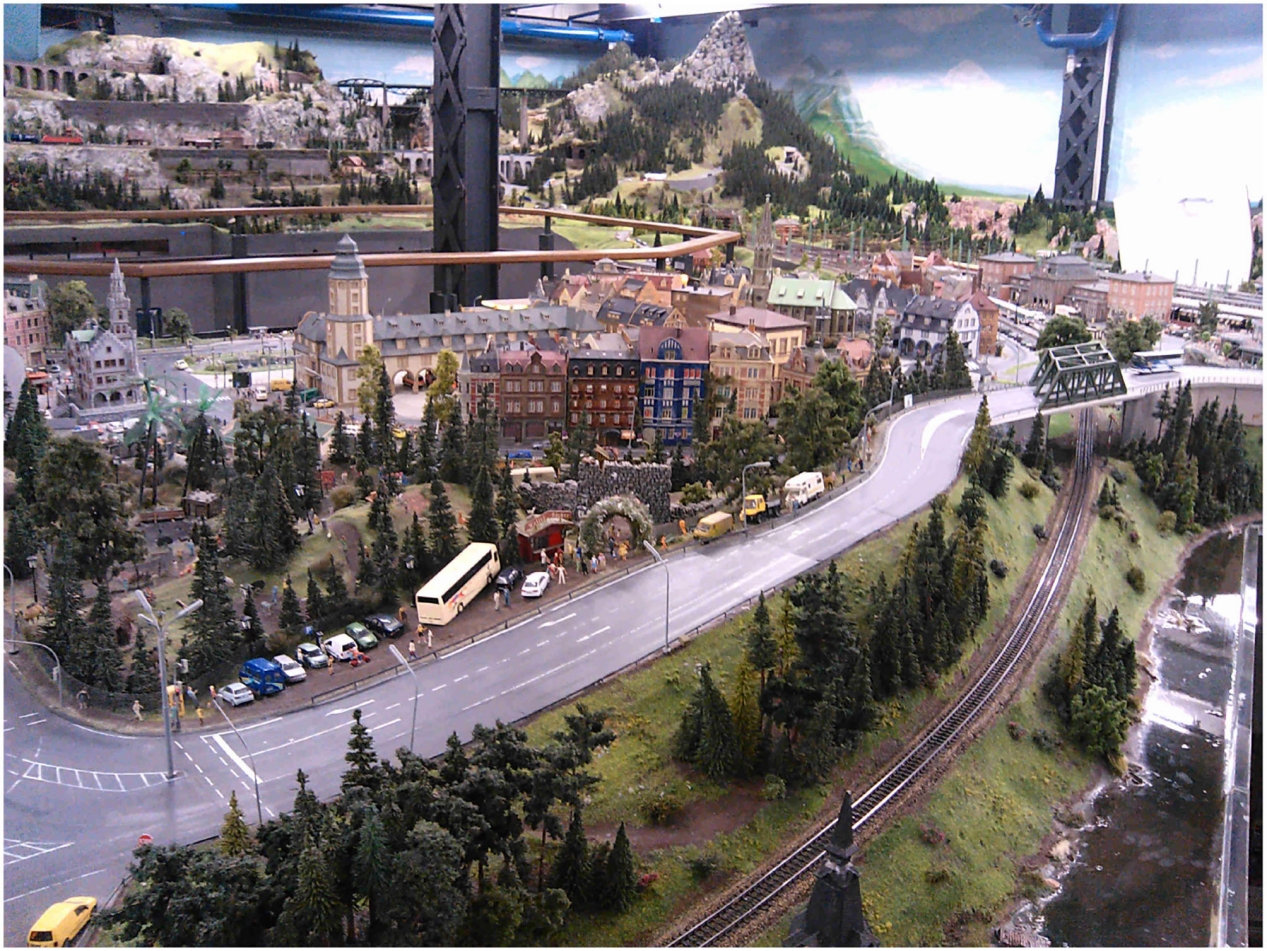
Sample view on the Miniatur Wunderland model railroad on a scale of 1:87. Parts of the 1,545 *m*^2^ layout are used for tests (Tiedemann, 2019; Wunderland, 2021, under CC-BY 4.0).

As the testing environment for longer test runs, the commercial 1:87 scale model railroad Miniatur Wunderland (1,545 m^2^ layout size, refer to Figure 2) is used (Wunderland, 2021). In the Miniatur Wunderland, different countries (with different kinds of traffic signs and road markings) are modeled. Furthermore, the lighting conditions can be changed to a night mode with street lamps and vehicle headlights switched on. So far we just collected data in a German street style area.

### 2.2. Miniature Road Vehicle

**Figure 3:**
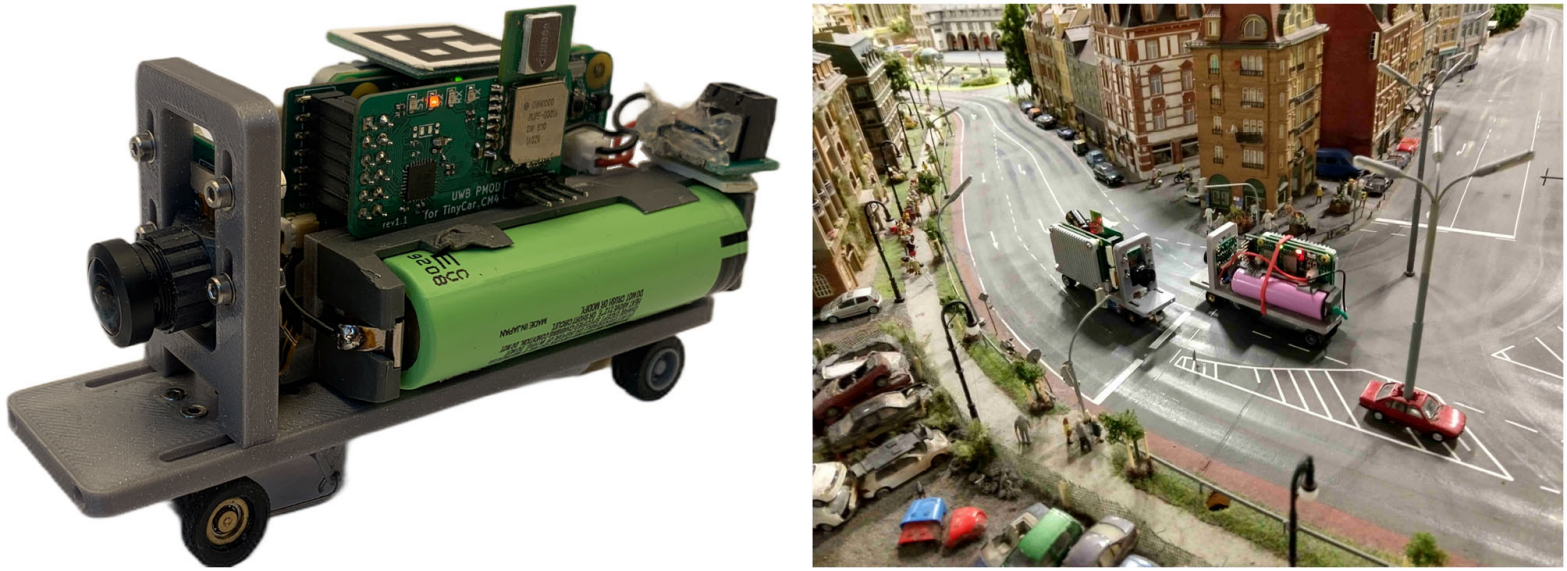
Left: Current version of the truck/van chassis used for the ADAS/AD algorithm design described in Section 4.3. Right: Two vehicles of the current van chassis design at the Miniatur Wunderland (backside of the part shown in Figure 2).

The current version of a model car that has sufficient computing resources for autonomous driving is based on our earlier versions, including a very compact sedan car, refer to Tiedemann et al. (2019). The TinyCar CM4 (refer to Figure 3) is the latest development of H0 scale (1:87) vehicles for autonomous driving in a miniature world. It is built around a powerful Raspberry Pi Compute Module 4 (CM4) as the main computing unit, with a custom PCB with two Coral Tensor Processing Units (TPU) providing up to 8 TOPS of machine learning power. Up to two cameras can be attached, with one front-facing, high-resolution ultra-wide-angle camera currently in use. An onboard IMU (BNO055) and an absolute encoder on the rear axle provide odometry data to supplement the localization information from an onboard ultra-wideband (UWB) indoor positioning system. A 3,500 mAh 18,650 Li-Ion battery provides power for a runtime of up to 3 h. All parts are on a 3D printed chassis. The total cost of a single vehicle is around 500 Euro.

All computational tasks are performed locally on the vehicle within a Robot Operating System (ROS) stack, making the vehicle truly autonomous and not reliant on any external hardware. With available local machine learning accelerators, even complex tasks such as image segmentation are computed directly on the vehicle. Using a robotic framework such as ROS improves the development and testing process a lot. The framework Rock/OROCOS from DFKI Germany / KU Leuven was discussed as an alternative but rejected because of the larger community of ROS.

The TinyCar CM4 is intended to be used in our own *Micro-Wunderland* and Hamburg's Miniatur Wunderland. The small scale makes it an ideal vehicle for autonomous driving education and research, as it poses no risks to researchers and their surroundings.

### 2.3. Miniature Aircraft

**Figure 4:**
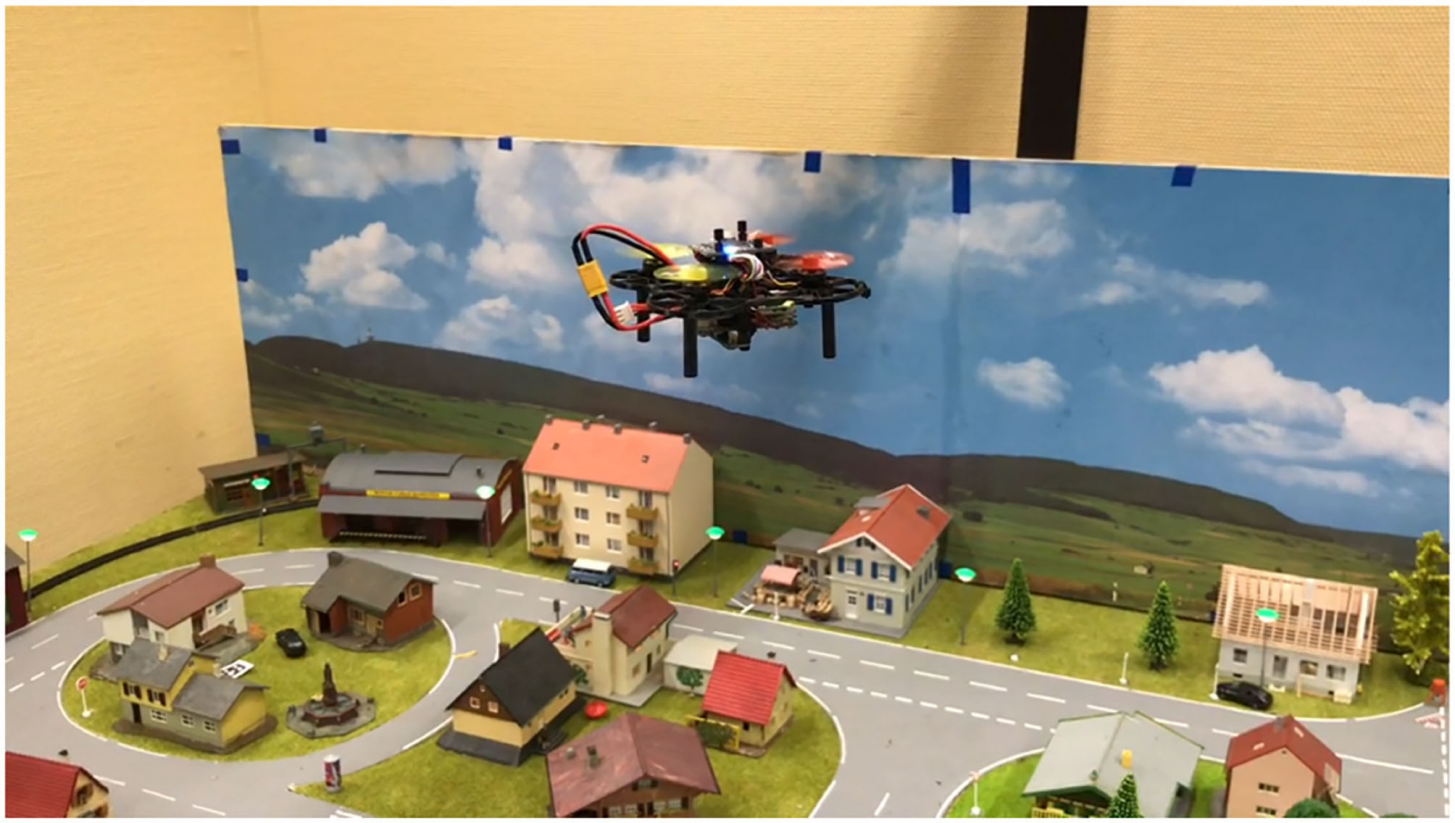
The micro air vehicle is flying over our small test area.

After a miniature ship and road vehicle, a micro air vehicle (MAV) was built to test and develop autonomous algorithms combined with visual object detection (refer to Figure 4). Our MAV is about 100 g and has a 3D-printed frame (10 × 10 cm). It has a flight time of 3–4 min. The hardware cost of one air vehicle is about 300 EUR. The platform uses a flight controller (Matek H743 Mini) and an AI Board (Sipeed Maix BIT) which can communicate by MAVLink protocol. An optical flow sensor is used for movement estimation over the ground. The optical flow is only stable in the short term, and the data will drift after some time. One option to correct this data over the long term is to use visual object detection.

To do this, a downward-facing camera will be attached to the MAV and connected to the AI board. An artificial neural network method can be applied to detect and classify markers such as cars, buildings, and roads in the environment. The goal is to have the MAV autonomously take off, fly, and land in the test area. Currently, the software is still under development, but flight and functionality tests were performed using a simple PID controller and a complementary filter for IMU data.

## 3. Method

### 3.1. Miniature Autonomy

The platforms described in the previous sections share a severe limitation in terms of space and weight. This leads to limitations in computing power and in the range of sensor devices that can be deployed. In order to find a solution for ADAS/AD tasks, we expect that at least in some cases down-scaling of existing solutions is not possible and new approaches have to be developed.

In the search for such new approaches, we must follow these three rules:

- All calculations (pre-processing of sensor data, environmental modeling and analysis, decision-making and control) must be performed on the vehicle itself. Data transfer to other systems is not permitted, except for remote control before/after testing or for emergency braking. This applies to the recall/inference phase of ADAS/AD solutions. For example, ML method training—when online learning is not part of the solution—may be conducted on other systems outside the vehicle. In cases where online learning is required as part of the solution on the later vehicle, this needs to be performed on the model vehicle, too.
- Only the sensors installed on the vehicle can be used.
- Only the power available on the vehicle can be used. However, we consider test runs to be successful when they last for a couple of minutes.

## 4. Results

The evaluation needs to be distinguished between a) the design of the hardware and software framework (the materials) i.e., the 1:87 scale platform, and b) the ADAS/AD design process that *uses* this platform and the results obtained in the application of this design process. We call the latter “algorithm under test (AUT)” and its design and results. The evaluation of the former is described at first.

### 4.1. Basic Function Tests

**Figure 5:**
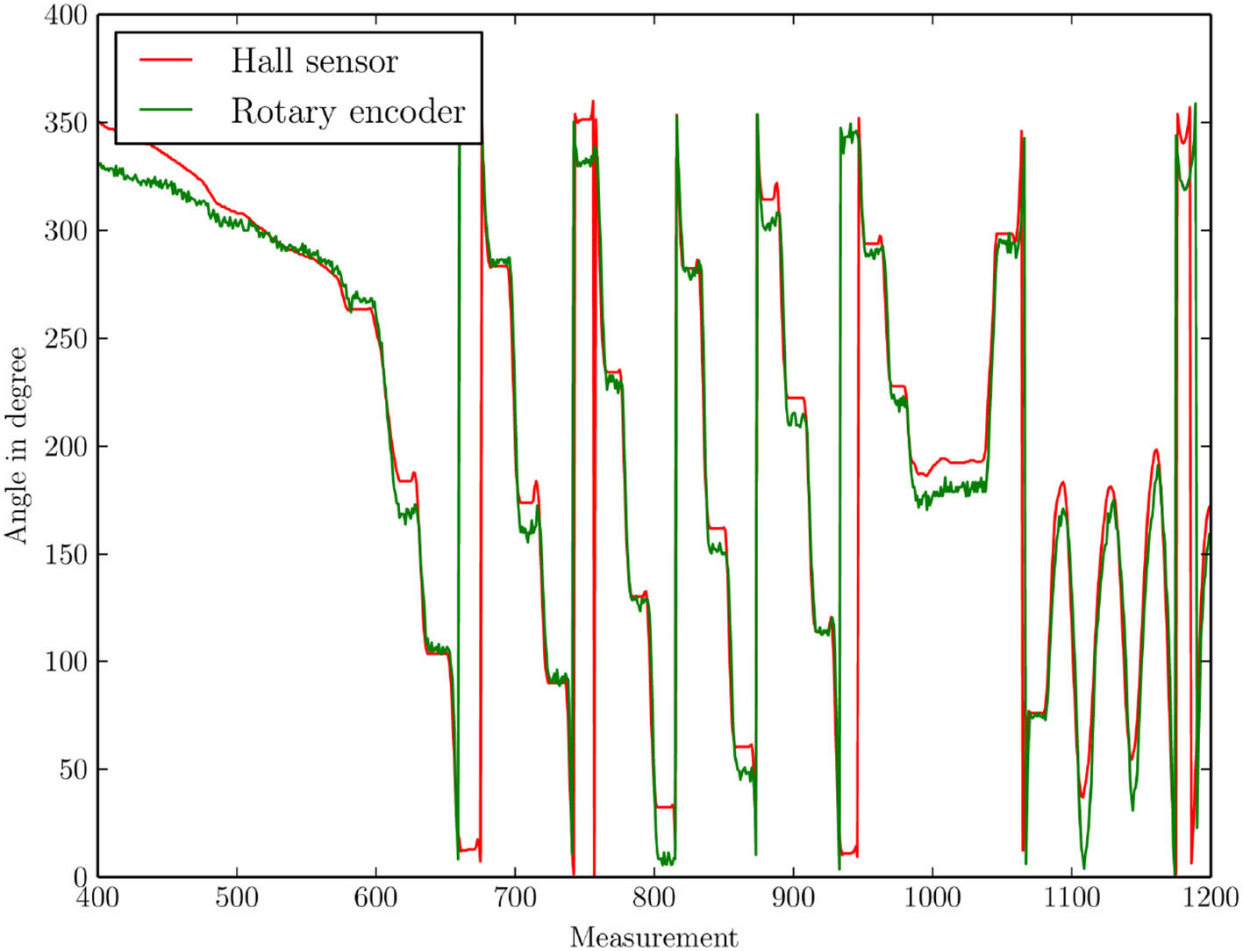
Comparison of the angles measured with the small-sized magnetic rotation encoder and the reference encoder (as ground truth).

Basic functional tests were performed with both vehicles presented (truck chassis and MAV). For both, a simple direct remote control was tested first, in which setpoints were specified for all degrees of freedom and simple control methods (PID, complementary filter) ran locally on the vehicle. For the truck chassis, a special focus is placed on the wheel encoder. Due to the small space available, a new solution had to be developed (refer to Section 3). An evaluation of the small magnetic encoder could be performed by attaching a (larger) reference encoder to the wheel (as ground truth) and comparing both encoder readings. A diagram of the comparison is shown in Figure 5.

The analysis of the data shows an average angular error of 6.9°. With a wheel circumference of 1.2 cm, this is an error of 0.23 mm per turn and 19.2 mm per driven meter. This can be improved but is sufficient for speed control and a rough odometry estimation.

**Figure 6:**
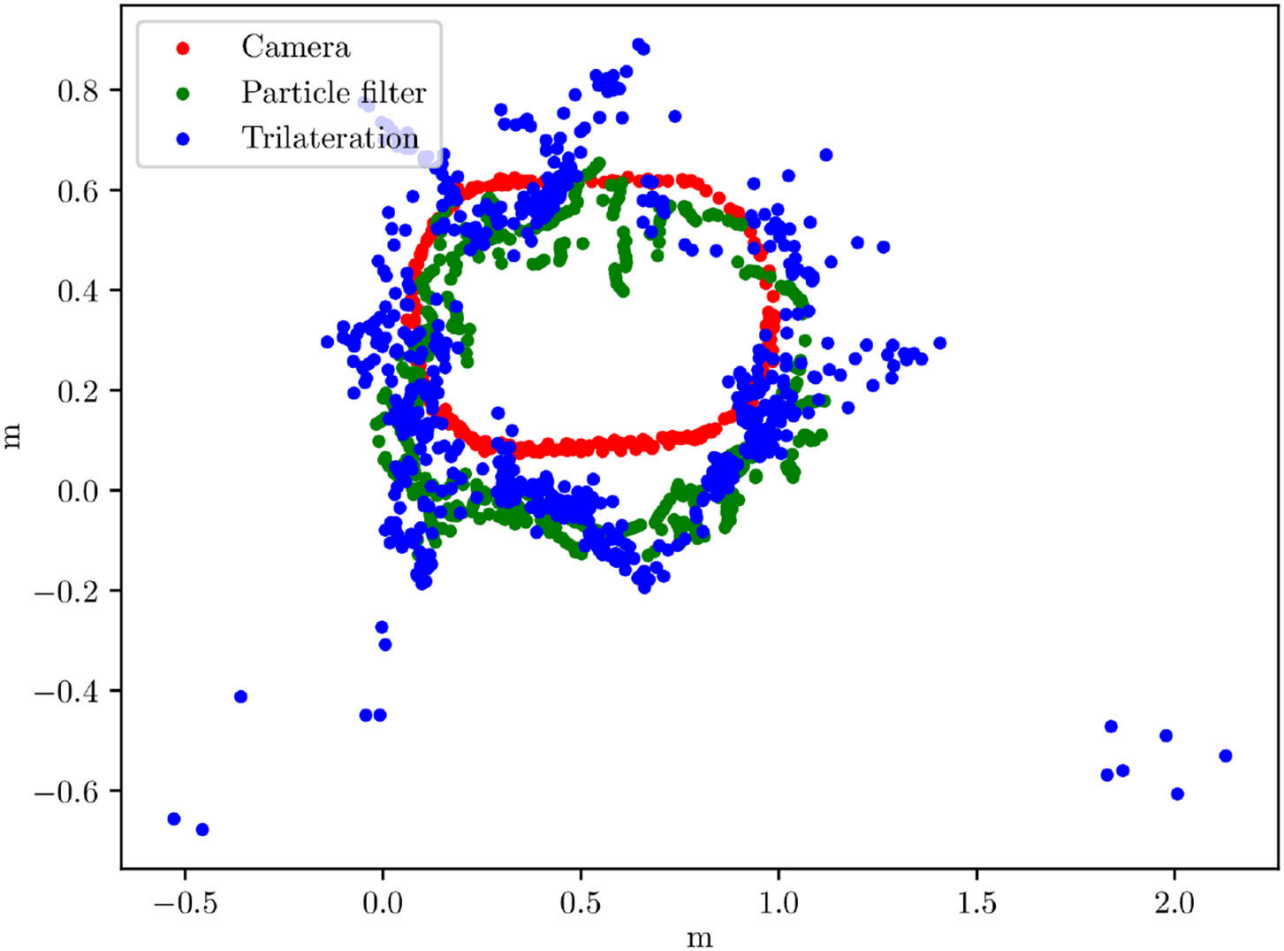
Plot comparing three localization methods developed for the small test area. Horizontal and vertical units are meters.

Another set of basic function tests examines the three localization methods. They have been developed for the small test area to serve as ground truth and to mimic GNSS-based localization. Localization is one central point in several ADAS and AD tasks, in lateral and longitudinal control. A comparison plot is given in Figure 6. Both UWB methods have parts with strong deviations from the camera localization (used as ground truth). The average error of the particle filter is 28.9 cm, and the average error of the trilateration method is 15.4 cm.

### 4.2. Miniature Autonomy Design Process: Scenario

To evaluate the miniature autonomy approach, a single typical autonomous driving (AD) task was selected as “algorithm under test (AUT)”: A road detection and tracking method was to be developed. Odometry, IMU, and onboard camera could be used as sensors.

### 4.3. Miniature Autonomy Design Process: Procedure

In developing the AUT, we initially started with the traditional approach. This consists of image processing to transform and filter the camera images and a CNN-based classification of the road segments in the images. During the design, several parameters needed to be adapted to the limited hardware (refer to below). Other constraints were imposed by the hardware and remained without any adjustments (e.g., the camera image resolution was 400 × 400 pixels at 8-bit gray values and 10 fps).

**Figure 7:**
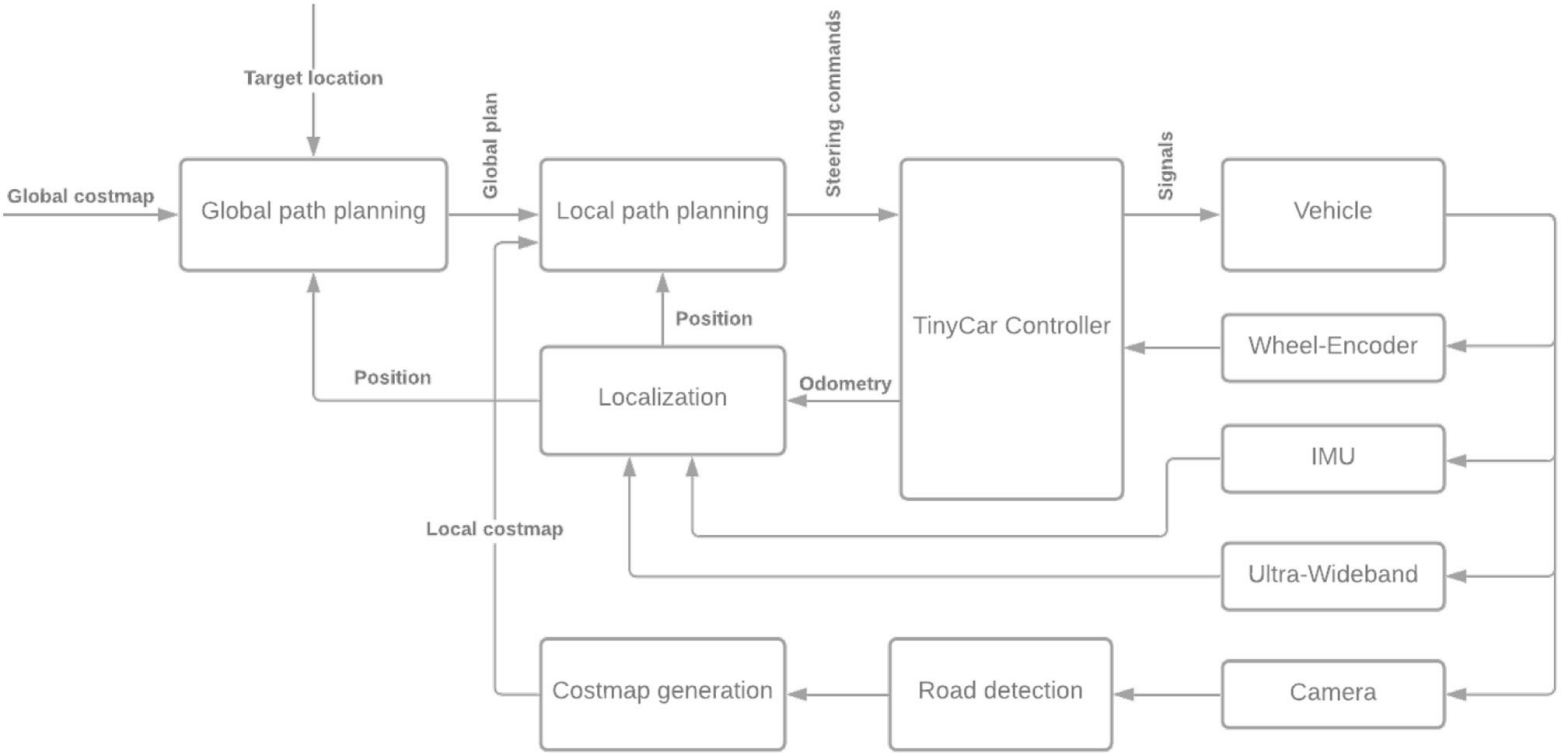
System architecture developed for the algorithm under test (AUT).

A diagram of the system architecture developed with the “miniature autonomy” constraints (the AUT) is shown in Figure 7. The nodes from left to right: The global cost map is generated with a static map. The global path is generated using a dwa_global_planner ROS node (move_base plugin). The local path planner is a slightly modified ROS teb_local_planner. The “TinyCar Controller” receives the planner's commands and generates steering and velocity control commands. It reads the wheel encoder data and generates the odometry. The localization is a ROS robot_localization. An extended Kalman Filter fuses odometry, UWB localization, and the IMU data. The road detection is done using a bilateral network with guided aggregation for real-time semantic segmentation (BiSeNet V2, Yu et al., 2020; Fan et al., 2021). In the “costmap generation” the detected road is transformed into a bird's view and written to the local map.

Road detection is central to the AUT and of particular interest for an evaluation of the “miniature autonomy” design approach. It is based on a BiSeNet V2 (Yu et al., 2020; Fan et al., 2021). However, to be computable on the EdgeTPU in the vehicle, the inference needs to be run with 8-bit unsigned integer values. Also, the TensorFlow Lite framework must be used if not all operations of the regular Tensorflow framework are available. The training was performed with standard floating point numbers. Then, the floating point numbers were quantized to uint8. Finally, since images of a rather coarse resolution can be used, the net architecture is also adapted (128 × 256).

As training data, three different data sets were used:

1. Small test area: In our small test area one data set with about 1,000 images was collected and manually labeled. The data collection was done using the truck chassis remotely controlled.
2. Carla: With the Carla simulator a second data set was collected including 1,500 images of the “Town02” map. This data set included different weather and lighting conditions.
3. CityScapes: The CityScapes data set was collected in 50 German cities and contains 4,000 pixel-wise segmented images (Cordts et al., 2016). Adding this data set, first, increases the training data size and, second, allows us to compare the effect of real-world data and artificial data and, third, fulfills a prerequisite to transfer results to the real world.

The selection and combination of different training data sets is an important point in machine learning (ML)-based solutions, also for combinations of simulations and real-world tests without miniature model tests: 1. most ML methods need the full variety of input data in the training data sets as it will occur later in the applications. 2. ML methods gain from large data sets, thus, each additional data set can improve the results. 3. A transfer from one to another training data set is an interesting test case in ML.

**Figure 8:**
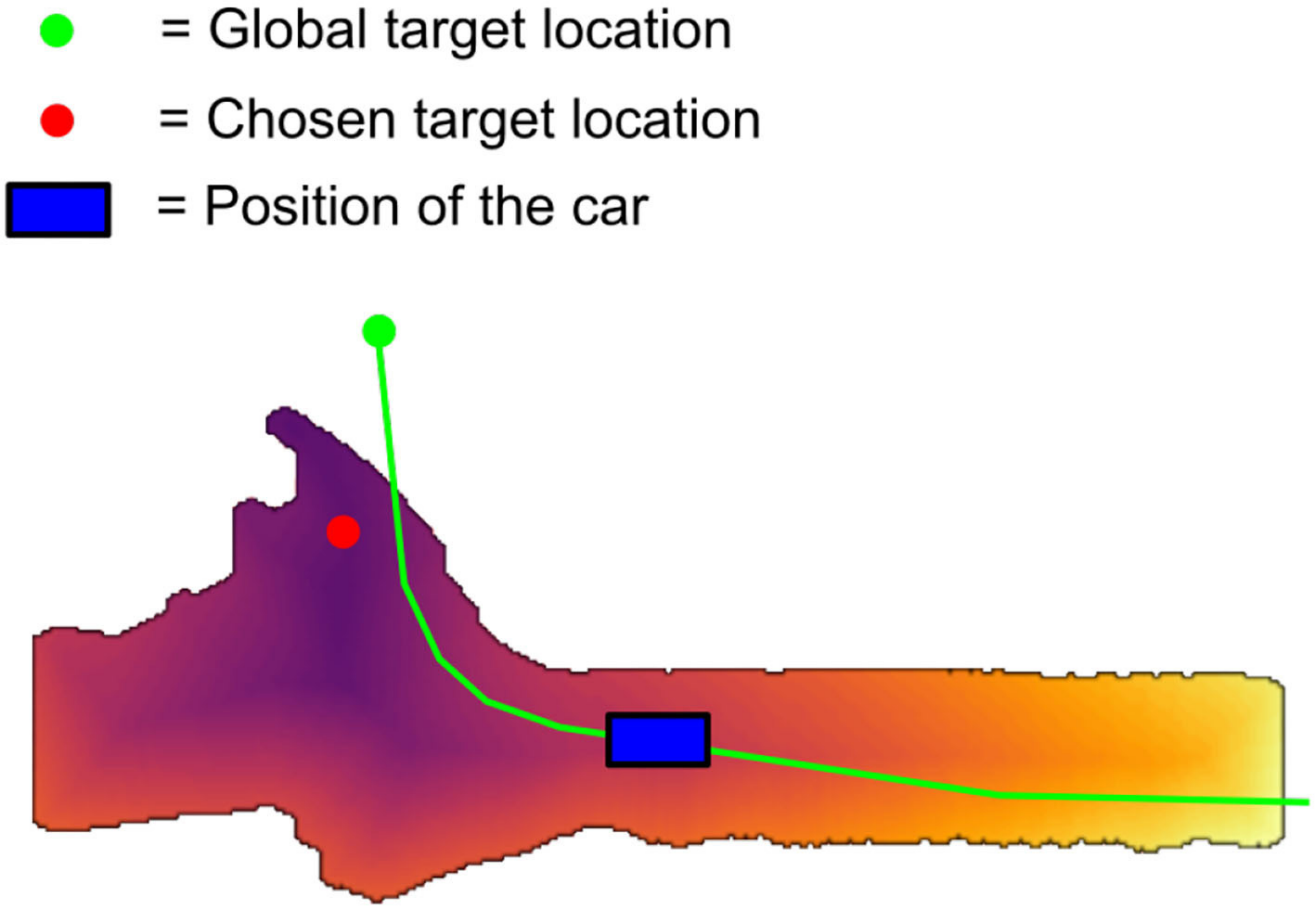
Visualization of the costs computed by the AUT.

An example of a cost map computed by the AUT is shown in Figure 8. For the given global target location a local target is chosen automatically within the (visible, computable) local map, using a method comparable to the proposal by Ort et al. (2019). Local chosen target and global given target usually differ as the local sensor data does not include the given global target at the beginning in most cases. However, the cost map computation is done dynamically while driving, thus, needs the current sensor data, and the local target needs to be reachable and on the local map. Details of the AUT design can be found in the thesis by Schwalb (2021).

### 4.4. Miniature Autonomy Design Process: Analysis of the AUT

Several tests were carried out with the AUT described above on the miniature truck and in the simulation. On the real vehicle on the small test site, several test runs had to be aborted without success. The reasons are a combination of a) the mechanical setup without damping or springs and with an imprecise steering mechanism, b) a suboptimal lens and visible ROI, and c) a suboptimal road classification. This often results in the inability to track the road and the vehicle leaving the road. A major improvement to correct this misbehavior is an optimized selection of training data. As another improvement, a new detection method is currently being implemented that includes lane-by-lane identification, refer to Section 6. However, the main purpose of this road following implementation is to serve as an AUT to investigate this design approach.

In about 10% of the runs, the entire path was traversed without such errors. This resulted in enough data to study the AUT behavior.

**Figure 9:**
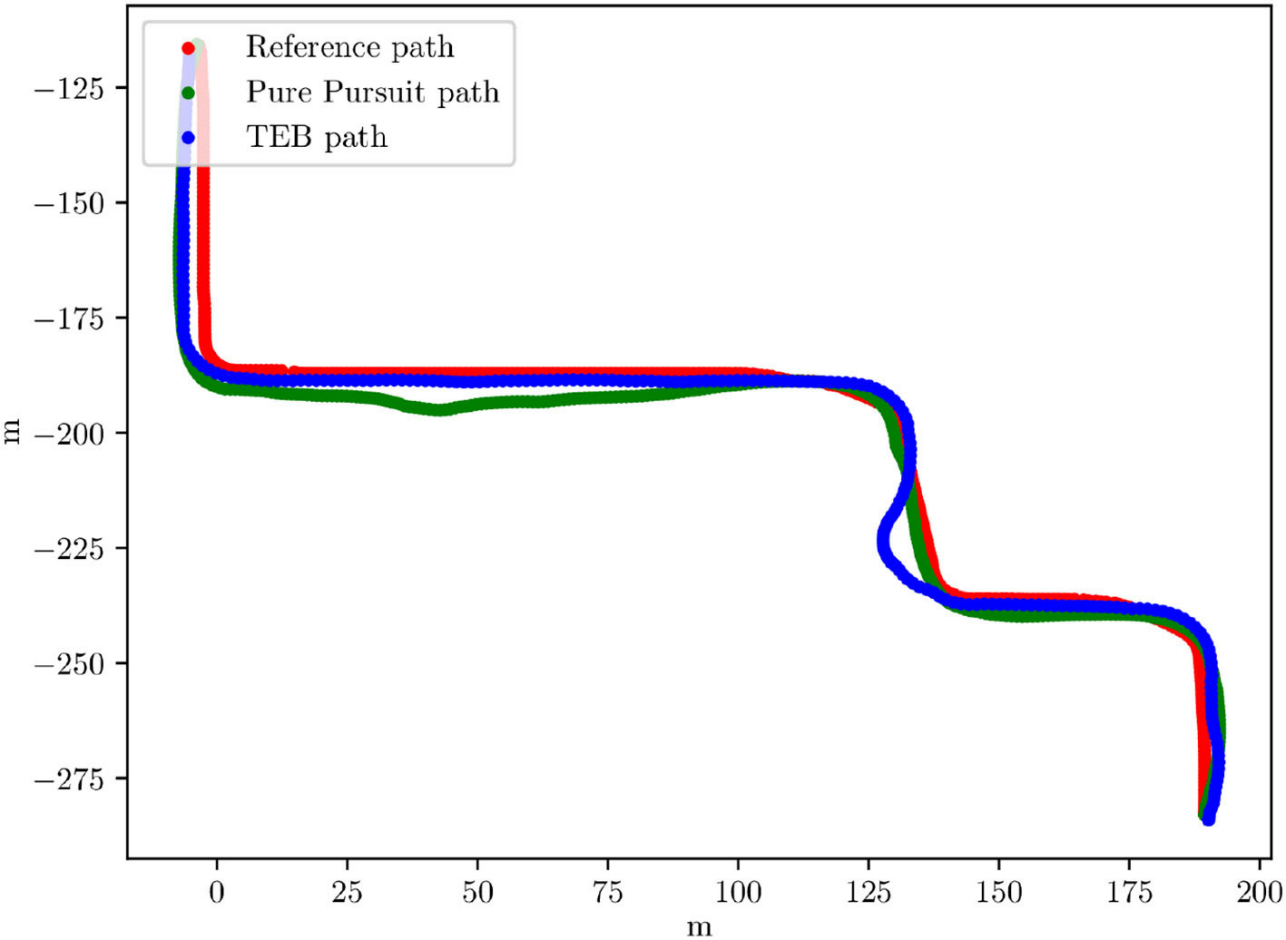
X/Y graph of a track driven in the Carla simulator with pure pursuit and with the TEB planner path. Given in red is the ideal reference path. The X/Y units are meters.

Figure 9 shows test runs in the Carla simulator. One test run shown uses the TEB local planner, one uses the pure pursuit planner. Both results are comparable, TEB has an average error of 2.0 m and pure pursuit of 3.1 m (in the Carla simulator the scale is 1:1, real traffic size). The large error in the TEB path is due to a street detection error. Comparing the behavior of the TEB planner and pure pursuit planner separately, it can be seen that the TEB planner has clear advantages.

**Figure 10:**
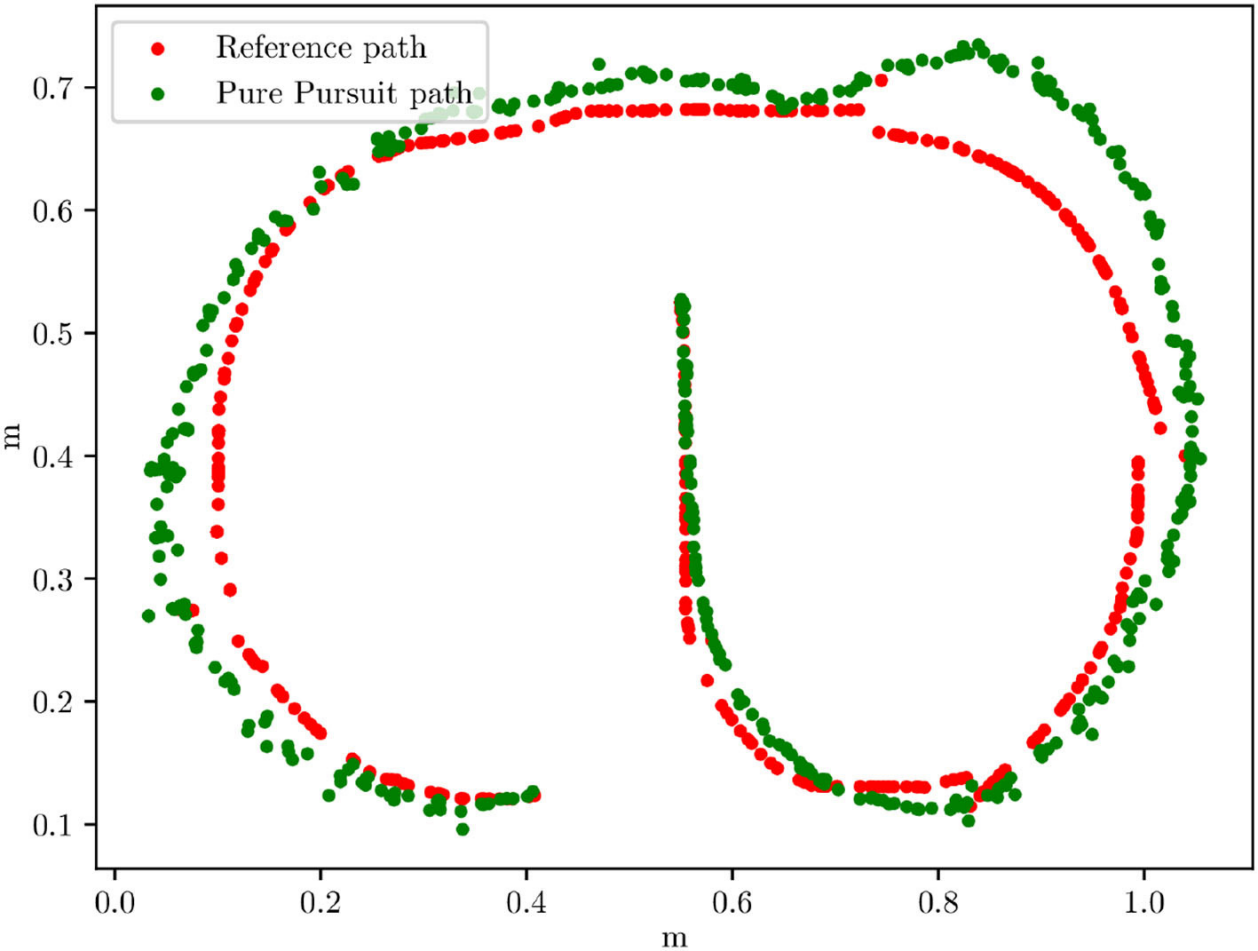
X/Y graph of a track driven with the real truck chassis and pure pursuit. Given in red is the ideal reference path.

Further test runs were performed on the miniature truck in the small test area. There, the system ran at a frame rate of 10 fps. In Figure 10 pure pursuit can be seen. The average error here is 0.029 m which, scaled by 1:87, is approximately 2.5 m, thus, comparable to the simulation results. In the overall behavior, it can be seen that the steering angle is always somewhat suboptimal. This can be explained by the inaccurate steering mechanics, which leads to a non-linear relationship between servo position and wheel angle.

So far no emulation of real-world drivers (other vehicles or pedestrians) was included since the simple automated (but non-autonomous) vehicles are currently developed and not ready yet (refer to Section 6). However, the purpose of the first study described here is to serve as AUT, thus, to study the miniature autonomy approach and to compare the solution of the AUT with a classical solution (refer to below).

### 4.5. Miniature Autonomy Design Process: Comparison With a Regular Design

The development of the AUT using the miniature autonomy approach resulted in a solution that differs in several ways from solutions published for real 1:1 scale vehicles.

In addition to the difference in hardware (e.g., computational hardware and sensors), the AUT implementation uses a downscaled BiSeNet V2 architecture, images with a coarse resolution of 400 × 400 pixels, and 8-bit gray values. The actual region of interest (ROI) is even smaller (300 × 300 pixels). Also, the inference calculation uses the Tensorflow Lite framework with a uint8 resolution instead of floating point values.

## 5. Discussion

In previous studies (refer to Schönherr, 2019), it was shown, that automated driving of a self-contained small scale vehicle in a well-defined environment is possible using only a microcontroller without an operating system and a small camera. The transition from automated driving to the development of semi-automated and autonomous driving on a small scale first requires a suitable environment. The Miniatur Wunderland in Hamburg and a specially developed small-scale test environment in our autosys research lab serve as such testing environments. Both environments exhibit a certain degree of complexity similar to real-world complexity and give rise to the designation *autonomous driving* as opposed to *automated driving*. The terminology *autonomous* is used here in the sense that the exact conditions under which the system is operated are not known at the time the algorithm is developed. Aspects of complexity include Changeable lighting conditions, configurable objects (houses, trees, parked cars, etc), traffic lights and street lighting, configurable roadway boundaries and conditions, and traffic.

Several platforms have been developed and tested over the years. It has proven beneficial to use the Robot Operating System (ROS) and incorporate Tensor Processing Units (TPUs) to take advantage of existing methods and apply machine learning methods for image recognition and feature extraction. However, non-ROS systems using NPUs (Neural Network Processing Units) as in our drone are also promising. In addition, various technological approaches have been developed and tested over the years, ranging from simple image feature extraction using OpenCV or self-implemented low-level methods, over several types of end-to-end approaches, to more complex methods using semantic segmentation and carefully adjusted steering controllers.

The current version of the miniature road vehicle described above is showing promise in terms of the choice of sensors, microcontrollers, electronics, and software. More work needs to be put into a smoother integration of the TPUs and more robust and precise mechanics of the steering subsystem. The final architecture regarding software and hardware of the miniature aircraft (drone) is still under investigation, although the first proof of concept was successful.

The miniature test environment has demonstrated some benefits as far as the overall concept is concerned. However, some robustness issues have recently become apparent, particularly in the design of the lanes. These are currently being redesigned to allow longer and more reliable test runs.

In summary, the miniature test environment could be used to test the “miniature autonomy design approach” and the AUT scenario of autonomous road detection and following has proven to be a viable test case. When comparing the AUT implementation on the miniature chassis with a standard implementation for real vehicles, some interesting differences can be observed:

1. A Raspberry Pi CM 4 together with a Coral EdgeTPU is a sufficiently powerful computation platform.
2. The small-size magnetic wheel encoder is a reliable solution with the potential option of increasing the resolution in the future.
3. An image resolution of 400 × 400 pixels (8-bit gray values) and a region of interest of 300 × 300 pixels (running at 10 fps) is enough for this task.
4. A BiSeNet V2 scaled down to 128 × 256 and inference computed with Tensorflow Lite (uint8) is sufficient as well.
5. Finally, the UWB localization showed sometimes “jumps” with single larger errors (refer to above). The particle filter solution could compensate and correct such errors.

However, there were hardly any qualitatively different solutions in this implementation—in particular, the BiSeNet V2 was just scaled down. This was different from a street-following implementation running on the sedan car (that used an ESP 32 without an ML hardware accelerator, Tiedemann et al., 2019). Nevertheless, we decided to use as much computational power as possible in the vehicles (including one system with an FPGA on its own PCB) to have the computational capacity to include more than just a street segmentation. These further projects with more complex tasks are already underway, refer to Section 6.

In this study, the purpose of the AUT was only to investigate the miniature autonomy design approach.

### 5.1. Limitations

While the proposed miniature autonomy design approach might lead to new solutions and to less needed test miles on real streets it does definitely not replace such real tests. The variety on real streets, with real environments, real other vehicles' behaviors, and real weather conditions are much larger than the variety within the miniature tests.

In the upcoming work, it needs to be checked if a quantitative measure in terms of standard benchmarks can be given to identify platform differences and to identify how much an introduction of miniature autonomy can change (refer to Section 6). Nevertheless, this will most likely be possible only for single parts, e.g., the behavior of the miniature vehicle mechanics, and not for the whole process including control, electronics, mechanics, and environment.

Furthermore, up to some extent, miniature sensors might be extended by a preprocessing step to mimic real sensors' properties by, e.g., adding noise, filtering, interpolation, delay, etc.). This will not be possible in all cases since physical properties differ quite a lot. The remaining differences between miniature and real-world systems (in sensor and/or actuator hardware) might cause an earlier occurrence of erroneous behaviors in miniature tests compared to real-world systems. Thus, it could uncover design errors. However, it might also be that errors experienced in the miniature model tests are caused by properties specific to miniature systems and, thus, are not relevant.

## 6. Outlook

The primary goal in the near future is to further stabilize the mechanics and electronics of our model-environment and miniature mobile platforms. In addition to study on robustness issues, the following projects are currently in progress or completed:

A sufficiently accurate positioning system is currently being developed using ultra-wideband technology and overhead cameras. This will allow quantification of test results, creation of a digital twin, synchronization with a simulation, and calibration of sensors.

A simulation environment of our model environment is being developed using Unity. This will allow the introduction of a simulation in the development process of AI related technology. A simulation will serve as another source of training data, enable the application of reinforcement learning methods, and provides a basis for investigating simulation-to-reality gap issues.

Currently, special *miniature smart cars* are being built. These will not steer autonomously but will drive along magnetic wires integrated into the roadway. Smart miniature cars will have some integrated sensors and WiFi capabilities. They will serve as smart traffic and as an additional source of data.

Further machine learning related questions are being investigated. More complex roadway and object segmentation methods (e.g., lane detection) are executed on the autonomous miniature platform with a focus on robustness issues. The special capabilities of our miniature test environment in terms of lighting conditions, traffic, changeable environment, etc. can be fully exploited.

Regarding the miniature autonomy design process and the study of this method our next steps are:

1. A comparison of the design process using small model tests vs. using simulations.
2. Tests with varying lighting conditions on the small test area and again the question of how this differs from simulations and if it is worth the additional effort.
3. An evaluation of “24/7” tests.
4. Finally a comparison of real vehicle tests vs. model tests.

## Data Availability Statement

The raw data supporting the conclusions of this article will be made available by the authors, without undue reservation.

## Author Contributions

TT and SP developed the theory and verified the analytical and experimental methods. TT conceived the proposal of the miniature autonomy design approach. LS, MK, and RG developed the software and hardware framework, performed and designed the data analyses and the evaluation. All the authors discussed the results and contributed to the final manuscript.

## Conflict of Interest

The authors declare that the research was conducted in the absence of any commercial or financial relationships that could be construed as a potential conflict of interest.

## Publisher's Note

All claims expressed in this article are solely those of the authors and do not necessarily represent those of their affiliated organizations, or those of the publisher, the editors and the reviewers. Any product that may be evaluated in this article, or claim that may be made by its manufacturer, is not guaranteed or endorsed by the publisher.
